# Navigating a Geriatrics Academic Career in Uncertain Times: Collective Resilience, Persistence, and the Value of Community

**DOI:** 10.1111/jgs.70440

**Published:** 2026-04-08

**Authors:** Maile A. Y. Karris, Liron D. Sinvani, Megan E. Young, Timothy W. Farrell

**Affiliations:** ^1^ Department of Medicine University of California San Diego San Diego California USA; ^2^ Northwell Health New Hyde Park New York USA; ^3^ Department of Internal Medicine, Section of Geriatrics Boston University Chobanian and Avedisian School of Medicine Boston Massachusetts USA; ^4^ Division of Geriatrics, Department of Internal Medicine Spencer Fox Eccles School of Medicine at the University of Utah Salt Lake City Utah USA; ^5^ VA Salt Lake City Geriatric Research, Education, and Clinical Center Salt Lake City Utah USA

**Keywords:** community, leadership, resilience

## 1 Introduction

Since March 2020, the healthcare landscape has been characterized by unprecedented crises, leading to persistent physician burnout. While challenges remain, these times have also catalyzed rapid advancements, including widespread telemedicine adoption [1], the integration of artificial intelligence (AI), and the Internet of Medical Things (IoMT) promising enhanced efficiency [2, 3, 4]. However, these innovations also bring concerns regarding effectiveness, data privacy, and unintended consequences [5]. Simultaneously, rapid shifts in U.S. policy, financing, and governance are reshaping healthcare [6, 7], raising critical questions for geriatricians: How can we sustain high‐quality, person‐centered care for older adults amidst such profound uncertainty? [8].

## 2 Insights From the Tideswell SIG Professional Development Session

Amidst this continuous change, the Tideswell Special Interest Group (SIG), in collaboration with the Tideswell Emerging Leaders in Aging program, hosted a symposium at the 2025 American Geriatrics Society Annual Meeting. This session aimed to empower colleagues by sharing strategies from leadership journeys in research, administration, clinical care, and education. Here, we summarize reflections from Tideswell SIG leaders on navigating a geriatrics career in uncertain times, including prompts (Box 1) to guide readers in identifying personal strengths and leadership strategies.

BOX 1 **Understanding your strengths: Self reflection.**

1. How do you manage fear or uncertainty during challenging times? What specific actions do you take when faced with adversity?
  - How do you typically process setbacks or failures?
  - What internal beliefs or values support your ability to keep going under pressure?
2. How do you maintain motivation when progress feels slow or invisible?
  - Who or what do you rely on during difficult times?
  - What strategies or routines have you developed to build your mental strength over time?
3. What have you learned about yourself through the challenges you've faced?
  - Has your definition of resilience changed over time? How so?
  - How do you measure your own growth in resilience or emotional strength?

## 3 Thrive in Your Context by Finding Your People and Cultivating Your Communities (Megan Young, MD)

Connection has always enriched my journey. From early research with Framingham Heart Study participants, whose stories resonated deeply, to my current home‐based primary care practice, I've understood the profound impact of human connection. Medical students accompanying me, sharing aspirations, offer vital hope amidst daily primary care challenges. My “Home Care Docs”—a micro‐community of seven women under 50, balancing careers, children, and leadership—are an essential support system. This group, along with other personal communities (e.g., parent groups, book clubs, running groups, family), provides constant advice, humor, and text‐accessible support. Building and maintaining these relationships requires effort, but the reward of belonging is immeasurable.

## 4 Nurture Your Career Growth by Finding Your Joy, Creating Opportunities, and Being Your True Self (Liron Sinvani, MD)

My path to geriatrics research was unexpected. As a third‐year resident, an unanticipated call opened the door to a fellowship. My mentor, Dr. Gisele Wolf‐Klein, transformed my career, fostering a research passion that yielded early publications and a state grant. However, pursuing NIH funding posed challenges: I lacked traditional research training, an NIH‐funded mentor, and an aging center affiliation. Prioritizing my institution and family, I forged a different path despite advice to relocate. The Tideswell ELIA program provided essential leadership skills and community. Empowered, I collaborated externally with Dr. Marie Boltz, a geriatrics nurse practitioner, who guided my first successful NIA grant. Her mentorship underscored that geriatrics, both clinical and research, thrives as a team sport. Since 2019, I've secured multiple grants, including an R01.

## 5 Employ Critical Strategies to Navigate the Perpetual Change and Uncertainty of an Academic Career (Timothy Farrell, MD, AGSF)

A 2016 Tideswell session, “What to do when the ground shifts beneath you,” proved prescient. Assuming a new leadership role, I experienced an early seismic shift with the unexpected departure of a key IPE program supporter. We adapted, assuming such disruptions were rare. By 2025, however, the ground shifts almost daily. My health system, like many, constantly updates to cope with the evolving healthcare landscape. My question evolved from “will the ground shift?” to “how, when, and where will it shift next?” As an Associate Chief, I now continually ask: “How do I navigate perpetual change and multiple crises, to not just survive but thrive?”

## 6 Ground Yourself and Deliberately Craft a Positive Community Culture (Maile Karris, MD)

During residency, an attending questioned my Infectious Diseases fellowship choice due to lower earning potential. My response was deeply personal: to honor my uncle's life. Growing up in Hawaii, I learned “ohana”—chosen family—with an open‐door home. Years later, learning my uncle died of AIDS, far from family, starkly contrasted that spirit. This revelation, coupled with HIV/LGBTQ+ stigma's impact, propelled me into a career focused on aging with HIV, honoring both those lost and those surviving. This is my “why.” Early in my assistant professorship, observing a competitive academic culture, I sought change. I invited three junior faculty to build a collaborative, supportive environment. Ten years later, we are thriving in our professional lives and continue to collaborate on grants and publications while navigating together life's struggles. Our collective strength surpasses individual efforts.

## 7 Conclusion

The Tideswell Emerging Leaders in Aging Special Interest Group co‐chairs have shared personal narratives demonstrating strategies for persistence and resilience (Table 1). We encourage colleagues to explore and refine their unique strengths (Box 1). Geriatricians are uniquely positioned to guide the healthcare community through uncertainty. Our expertise in team‐based, person‐centered, and interprofessional care equips us to champion holistic, equitable systems [9]. Please see Supporting Information for leadership development opportunities in Aging and Geriatrics.

**TABLE 1:** Strategies for persistence and resilience.

Themes	Key lessons learned
Cultivating Community	**Lesson 1: Proactively Build and Nurture Your Network.** Creating and maintaining a strong community takes consistent effort, but the returns in support, advice, and resilience are invaluable. If you're not part of a close‐knit group, initiate one. **Lesson 2: Align with a Shared Mission.** Communities thrive when members share a common purpose. Staying true to our collective goal of delivering excellent care to older adults in the community strengthens our bonds and fuels our perseverance. **Lesson 3: Embrace Mutual Support.** Rely on your community for both professional and personal uplift. The shared humor, insights, and emotional backing can be a powerful antidote to challenging days.
Nurturing Career Growth	**Lesson 1: Be Open to Opportunity, Yet Authentic.** Thriving emerges from a blend of hard work, seizing unexpected opportunities, maintaining a positive outlook, and, crucially, staying true to your values and personal circumstances. **Lesson 2: Surround Yourself with Believers.** Cultivate relationships with colleagues, mentors, and communities who champion your potential, especially when self‐doubt creeps in. Their unwavering belief can be a powerful motivator. **Lesson 3: Practice Respect, Support, and Kindness.** As Donna Fick wisely advises, these principles are fundamental to a fulfilling career. Authenticity, honesty, loyalty, and diligent effort, combined with a touch of luck, are the bedrock of success.
Employing Critical Leadership Strategies	**Lesson 1: Prioritize and Strategize in Crisis.** Not every crisis demands immediate attention, nor can it always be addressed at once. Like a firefighter, assess which “fires” are most critical and where your often‐limited resources will be most effective. Ask: “Will this crisis fade, or grow rapidly?” and “Am I the right person to lead this, and who else on the team should be involved?” **Lesson 2: Communicate Effectively with Empathy.** In times of crisis, clear and empathetic communication is paramount. Adopt a strategy of being first with information, ensuring its accuracy (“Be Right”), expressing empathy, promoting actionable steps, showing respect, and demonstrating transparency. **Lesson 3: Embrace Crises as Opportunities for Growth.** View difficult situations not as setbacks, but as catalysts for positive change and development. The adage “never let a crisis go to waste” holds true. Reflect on what new opportunities might arise when old paths are blocked, asking, “what can I now pursue that I couldn't before?”
Grounding Yourself and Building a Positive Culture	**Lesson 1: Re‐center by Reconnecting with Your “Why.”** In overwhelming or uncertain times, remembering your core motivation for doing this work provides fortitude. Your dedication is meaningful, makes a difference, and brings joy; anchoring to this “why” empowers continuous progress. **Lesson 2: Actively Cultivate a Supportive Community.** While scarcity and uncertainty might tempt isolation, investing in your professional community fosters collective strength. True thriving comes from mutual support and advocacy, making everyone stronger together. **Lesson 3: Be the Change You Wish to See.** If you observe a cultural norm that isn't conducive to thriving, take the initiative to create a different environment. A few dedicated individuals can transform a competitive landscape into a collaborative one.

## Author Contributions

All authors listed significantly contributed to the conceptualization, design, and preparation of the manuscript.

## Disclosure

There was no sponsor for this manuscript. Nancy Lundebjerg initially conceptualized the program. Drs. Karris, Sinvani, Young, Farrell, Chang, Walter, and Ritchie developed the purpose, content, and program, presenting the content at AGS 2025. Drs. Karris, Sinvani, Young, and Farrell participated in the writing and editing of this publication.

## Consent

We have obtained written consent from additional contributors, not listed as authors, who are named in the acknowledgments section.

## Conflicts of Interest

The authors declare no conflicts of interest.

## Supporting information


**Table S1:** Leadership development opportunities in aging and geriatrics.
