# Supplementary material for: Navigating a Geriatrics Academic Career in Uncertain Times: Collective Resilience, Persistence, and the Value of Community
Source: J Am Geriatr Soc. 2026 Apr 8;74(7):2149–51. doi: 10.1111/jgs.70440 (PMC13418676; doi:10.1111/jgs.70440)
Supplement: Supplementary file 1 — Table S1: Leadership development opportunities in aging and geriatrics. [file JGS-74-2149-s001.pdf]

Supplemental Table 1. Leadership Development Opportunities in Aging & Geriatrics			
**Category**	**Program/Opportunity Name**	**Description/Purpose**	**Target Audience**
Formal Leadership Development Programs	AGS/ADGAP Leadership and Life Skills Development	Provides a curriculum focused on leadership and life skills for those committed to a career in academic geriatrics.	AGS Fellows-in-Training (FITs), early-career professional members
Formal Leadership Development Programs	Tideswell Emerging Leaders in Aging (ELIA)	A year-long program focusing on clinical, education, and research leadership to shape the future of healthcare and social systems for older adults.	Advanced junior or mid-career clinicians, educators, or researchers with a clear commitment to the field of aging
Formal Leadership Development Programs	Leadership Training for Palliative Care and Geriatric Professionals (CAPC)	Specific leadership training offered by the Center to Advance Palliative Care (CAPC), often focused on program development, team management, and strategic planning in palliative care, with significant overlap for geriatrics.	Organizations that are members of CAPC, often targeting program leaders, medical directors, and emerging leaders in palliative and geriatric services
Research-Focused Leadership (NIH/NIA)	NIA Butler-Williams Scholars Program	An intensive program designed to introduce new investigators to aging research, including opportunities for scientific networking and career development planning.	Early-career researchers (junior faculty, postdocs) from underrepresented groups, or those new to aging research
Research-Focused Leadership (NIH/NIA)	NIH Career Development Awards (K Awards)	Provide mentored research career development for individual investigators, fostering leadership of independent research programs and often including dedicated professional development plans (e.g., K08, K23, K01).	Early to mid-career faculty focused on establishing independent research careers
Research-Focused Leadership (NIH/NIA)	NIA-Funded Training Grants (T32, R25)	T32s fund institutional research training programs; R25s support research education programs. Faculty leading these grants gain experience in program management and mentorship, while participants develop research leadership skills.	Faculty (as program directors/mentors); pre- and post-doctoral trainees (as participants)
Research-Focused Leadership (NIH/NIA)	NIA Centers (ADRCs, Pepper Centers)	Large, multidisciplinary research centers (e.g., AD/ADRCs, Pepper Centers) often feature mechanisms to support junior investigators and provide leadership roles for senior faculty in managing cores or scientific initiatives.	Faculty (junior and senior) involved in aging research; offers roles in core leadership, committee service, pilot program direction
Research-Focused Leadership (NIH/NIA)	HRSA Geriatrics Workforce Enhancement Programs (GWEPs)	Federal grants that fund institutions to improve care for older adults by developing a skilled healthcare workforce. Leaders manage interprofessional teams, design curricula, and implement community outreach programs.	Institution-based faculty and program leaders focused on interprofessional education, workforce development, and clinical innovation in geriatrics
Professional Society Roles	Gerontological Society of America (GSA)	Offers numerous leadership opportunities including section leadership (e.g., Behavioral and Social Sciences, Health Sciences), Special Interest Group (SIG) roles, journal editorial board service, and engagement with the Emerging Scholar and Professional Organization (ESPO).	Researchers, educators, practitioners, and policymakers across disciplines related to aging; ESPO for early-career professionals
Professional Society Roles	American Academy of Hospice and Palliative Medicine (AAHPM)	Provides leadership roles in committees, Special Interest Groups, and elected board positions for professionals in hospice and palliative medicine, often overlapping significantly with geriatrics.	Palliative care and geriatric medicine physicians and other professionals

Professional Society Roles	AMDA - The Society for Post-Acute and Long-Term Care Medicine	Offers leadership in committee involvement, advocacy, clinical practice guideline development, and educational initiatives for professionals in post-acute and long-term care settings.	Medical directors and attending physicians in long-term care settings (e.g., SNFs, ALFs)
Professional Society Roles	American College of Physicians (ACP) / Society of General Internal Medicine (SGIM)	Both societies have geriatrics-focused committees or sections, providing leadership opportunities in policy advocacy, education, and professional development within a broader internal medicine context.	Internists, including those with a focus on geriatrics
Philanthropic & Advocacy Initiatives	The John A. Hartford Foundation Initiatives	Funds large-scale initiatives aimed at improving care for older adults. While direct fellowships may vary, their grant programs often include leadership development components or seek leaders to champion their efforts.	Clinicians, researchers, and educators committed to improving geriatric care; individuals seeking to drive systemic change
Philanthropic & Advocacy Initiatives	Disease-Specific Foundations	Organizations like the Alzheimer's Association, American Heart Association, or American Cancer Society often have research and advocacy arms focused on older adults, offering leadership roles on scientific panels, grant review committees, and public advocacy efforts.	Researchers and clinicians specializing in age-related diseases; those interested in advocacy and policy influence
Institutional & Local Leadership	Academic Leadership Roles	Positions within university departments such as Division/Section Chief, Fellowship/Residency Program Director, Clerkship Director, or Course Director. Also includes leadership of dedicated aging centers or institutes.	Faculty at all career stages; individuals seeking to lead academic programs, divisions, or centers
Institutional & Local Leadership	Hospital/Health System Leadership	Roles like Medical Director for specialized geriatric programs (e.g., Geriatric Fracture Program, ACE Unit), or broader hospital leadership roles such as Chief of Staff, Department Chair, or Chair of quality/safety committees.	Geriatricians interested in system-level leadership, quality improvement, and clinical program development
Mentorship & Development	Mentorship Programs	Serving as a mentor to junior faculty or trainees is a fundamental leadership role, fostering the next generation of geriatrics professionals. Participating as a mentee in structured programs also builds future leaders.	Experienced faculty (as mentors); junior faculty and trainees (as mentees)
Policy & Government Leadership	National Academy of Medicine (NAM) / NASEM	Serving on prestigious committees that generate influential reports on health policy, research directions, and workforce needs related to aging. Membership in NAM is a high-level recognition of leadership.	Senior leaders, experts, and policymakers in health and aging
